# Identification and characterization of a novel infectious bursal disease virus from outbreaks in Maharashtra Province of India

**DOI:** 10.14202/vetworld.2018.1516-1525

**Published:** 2018-10-29

**Authors:** Sudhakar P. Awandkar, Prabhakar A. Tembhurne, Jeevan A. Kesharkar, Nitin V. Kurkure, Sandeep P. Chaudhari, Sachin W. Bonde, Vijay C. Ingle

**Affiliations:** 1 Department of Veterinary Microbiology, Nagpur Veterinary College, MAFSU, Nagpur - 440 006, Maharashtra, India; 2 Department of Veterinary Pathology, Nagpur Veterinary College, MAFSU, Nagpur - 440 006, Maharashtra, India; 3 Department of Veterinary Public Health and Epidemiology, Nagpur Veterinary College, MAFSU, Nagpur - 440 006, Maharashtra, India; 4 Department of Veterinary Biochemistry, Nagpur Veterinary College, MAFSU, Nagpur - 440 006, Maharashtra, India

**Keywords:** adaptation, chicken embryo fibroblast, epidemiology, isolate infectious bursal disease virus, VP_2_

## Abstract

**Aim:**

The study was undertaken to isolate infectious bursal disease virus (IBDV) from clinical cases in broiler and cockerel flocks of Maharashtra state, India, and its molecular epidemiological investigation.

**Materials and Methods:**

The morbid bursal tissues were collected from flocks suspected for IBD. The samples were subjected for virus adaptation in primary chicken embryo fibroblast (CEF) cells followed by confirmation by reverse transcription polymerase chain reaction (RT-PCR) for partial VP_2_ sequence and phylogenetic analysis.

**Results:**

The isolation of IBDV from field samples took seven blind passages for adaptation in CEF. The cytopathic effects included rounding, aggregation, vacuolation, and detachment of the cells. The RT-PCR showed amplification of 627 bp amplicon specific to the primers for VP_2_ gene fragment which confirmed successful adaptation and isolation of IBDV using CEF. The nucleotide and deduced amino acids based on phylogeny clustered the current isolate in a distinct clade with classical virulent and antigenic variants. It showed divergence from very virulent (vv) and vaccine strains of Indian origin. The isolate showed unique amino acid substitution at A329V as compared to all other IBDVs. The variation in key amino acids was reported at A222, I242, Q249, Q253, A256, T270, N279, T284, I286, L294, N299, and V329. It shared conserved amino acids at position A222, I242, and Q253 as reported in vvIBDV isolates. However, the amino acids reported at position T270, N279, T284, L294, and N299 are conserved in classic, antigenic variant and attenuated strains of IBDV. The amino acids at positions N279 and T284 indicated that the isolate has key amino acids for cell culture replication.

**Conclusion:**

The IBDV field isolate does not reveal the full nucleotide sequence signature of vvIBDV as well as vaccine strains. Hence, we can conclude that it might not belong to vvIBDVs of Indian origin and the vaccine strain used in the region. This may be suggestive of the evolution of the IBDV in the field due to the coexistence of circulating field strains and live attenuated hot strains, resulting into morbidity and mortality, warranting the need for safer protective vaccines, and implementation of stringent biosecurity measures to minimize loss to farmers.

## Introduction

The infectious bursal disease (IBD) is a highly contagious viral disease of chickens. The disease was emerged in 1957 [1] in Gumboro, USA. Chickens of the age group of 3-6 weeks are most susceptible to clinical disease. At this stage, maternal immunity disappears, and immature B cells populate the bursa of Fabricius. Successful infections followed by an incubation period of 3-4 days lead to a clinical disease characterized by high morbidity, mortality, and marked immunosuppression [2]. The severity of the immunosuppression depends on the virulence of the virus and age of the host. In naïve birds, the mortality may reach up to 100%. The necropsy indicates swollen, edematous, yellowish, and occasionally hemorrhagic bursa, especially in birds that died of the disease. In addition, hemorrhage of the pectoral and leg muscles can also be seen. The massive destruction of bursal follicles is observed along with the lack of regeneration. Therefore, recovered chickens from isolate IBD virus (IBDV) infections show small, atrophied cloacal bursa. The broiler weight gain is delayed by 3-5 days. The IBD incurs huge direct and indirect economic losses to the poultry industry and poultry farmers worldwide [3,4]. The causative agent, IBDV, is a non-enveloped virus with bisegmented double-stranded ribonucleic acid (dsRNA) genome belonging to family *Birnaviridae* and genus Avibirnavirus [5]. The virus has two serotypes. The IBDV serotype 1 (classic and variant) causes disease in chickens, and the antigenic variation can exist between strains. Although antigenic variation occurs through antigenic drift, genome homologous recombination can also contribute to it [6]. IBDV serotype 2 strains infect chickens and turkeys but have not caused clinical disease or immunosuppression [7]. After the emergence of classical IBDV in 1957, it had rapidly spread throughout the USA, Europe, Asia, and other parts of the world in an explosive manner but has not yet been reported in New Zealand [8-11]. The Australian field strains are considered as low pathogenic causing subclinical disease with immunosuppression [12]. The disease is endemic in parts of Southern Asia including India, Indonesia, South America, Middle East, and Africa [13]. It has been clinically reported in 80% member countries of the World Organization for Animal Health [14].

Due to its highly infectious nature and resistance to inactivation, strict biosecurity measures are needed with vaccination in a high-risk population. It also becomes essential to protect chicks at the early age group. Most commercially available conventional live IBDV vaccines are based on classical virulent strains. The vaccines constituting mild strains exhibit poor efficacy, while the “Intermediate” and “intermediate plus” or “hot” vaccines have much better efficacy and may break through higher levels of maternally derived antibodies [15]. However, these vaccines are seen to induce moderate-to-severe bursal lesions and thus cause corresponding levels of immunosuppression [16] with low mortality. They may not fully protect chickens against infection by the very virulent IBDV (vvIBDV) strains [17]. In spite of routine vaccination, regular outbreaks have been reported frequently from different parts of the world due to the antigenic variations in the IBDV genome [18]. Second, the safety and efficacy of available vaccines still remain a major concern.

The present study was planned with the objective to isolate and characterize the circulating IBDV from mortalities reported in broiler and cockerel flocks of Maharashtra Province of India using molecular epidemiological tools.

## Materials and Methods

### Ethical approval

The present study was conducted after the approval of the Board of Studies and the Institutional Animal Ethics Committee.

### Samples

The samples (n=27) were collected from the birds aged 3-6 weeks of age suggestive of IBD from nine suspected outbreaks in Maharashtra Province of India during the year 2014-2016. The samples constituted edematous and hemorrhagic bursae from morbid cockerel and broiler birds exhibiting clinical and gross pathological features of the disease. The bursal tissues were collected aseptically in pre-sterilized ice-chilled screw cap vials with minimum essential medium (MEM) from morbid chickens and transported to the laboratory on ice.

The bursal tissue was weighed, triturated in pre-sterilized frozen pestle and mortar, and minced in fine powder to obtain 10% suspension in sterile normal saline. This suspension was subjected to three cycles of freezing and thawing. Then, the suspension was clarified at 10,000 rpm for 10 min in a refrigerated centrifuge. The supernatant was collected and filtered through a 0.22 μm syringe filter. The filtrate was stored at −80°C until further use.

### Isolation of virus

The 9-day-old embryonated eggs were obtained from Central Hatchery, Nagpur. The single cell suspension of the fibroblasts was obtained by mincing and trypsinization at 37°C. The cell pellet was resuspended in MEM (Sigma, USA) supplemented with 10% Fetal Bovine Serum (Gibco BRL, USA). The live cell count was adjusted to approximately 6 million cells/ml, dispensed in the wells of 24-well tissue culture plates, and incubated at 37°C with 5% CO_2_ for 12 h in a CO_2_ incubator (Thermo, USA) to get approximately 80–85% confluence [19].

The supernatant medium from a confluent monolayer of the attached fibroblasts was discarded, and the monolayer was washed with cell culture grade phosphate buffer saline. The cells were infected with 50 μl of inoculum per well in duplicate. The plates were incubated at 37°C in a CO_2_ incubator for 1 h with intermittent mixing after every 10 min. Two wells were kept as uninfected control while two were infected with commercially available vaccine virus (Ventri-Plus, India). The maintenance medium was added and incubated at 37°C in a CO_2_ incubator. The cells were observed twice daily for the development of cytopathic effects (CPEs). The samples were subjected to blind passages until the CPEs were observed. The cells showing CPEs were stored at −80°C until further use.

### Identification

The isolates were confirmed by reverse transcription polymerase chain reaction (RT-PCR) technique.

For molecular identification, the total RNA from cells showing CPEs was extracted using TRI reagent (Sigma, USA) [20]. The RNA pellet was dissolved in 20 μl diethyl pyrocarbonate-treated water followed by differential precipitation using lithium chloride [21]. The viral dsRNA was quantified using Nanodrop 1000 (Thermo Fisher, USA). The viral dsRNA was used as a template for synthesizing complementary deoxyribonucleic acid strands using SuperScript III First-Strand Synthesis System for RT-PCR (Invitrogen, Catalog No. 18080–051) as per manufacturer’s instructions. Briefly, 100 ng of dsRNA was taken for cDNA synthesis. To this, random hexamer (50 ng) and dimethyl sulfoxide (2 μl) were added in 10 μl reaction. The reaction mixture was incubated at 90°C in a thermal cycler for 5 min and snaps chilled in ice. To this, 10 µl of RT mix (10 mM dNTPs, ×10 RT buffer, and 200 u reverse transcriptase) was added to form 20 μl reaction. The reaction was incubated at 25°C for 10 min, 37°C for 120 min, and 70°C for 5 min.

The cDNA was subjected for PCR using designed primers (VP_2_F- ACTGTCCTCAGCTTACCCACAT, VP_2_R- TCTGTGACCAGGTTCTTTGCTA) targeting amplification of VP_2_ structural gene of IBDV [22]. The PCR was carried out at 94°C for 3 min, followed by 35 cycles of denaturation for 30 s, annealing at 49°C, and extension at 72°C for 1 min each followed by final extension for 10 min. The PCR amplicons were confirmed by electrophoresis in 1% agarose gel (with ethidium bromide) in 0.5 × (Tris-borate-ethylenediaminetetraacetic acid buffer [21]. The agarose gel was carefully removed, and imaging was done using a gel documentation unit (G-Box, Syngene).

### Sequencing and phylogenetic analysis

The RT-PCR product from tissue culture adapted field IBDV was purified using QIA quick gel extraction kit (Qiagen Cat. No. 28704) following manufacturer’s instructions. The gel purified amplicon was subjected for commercial sequencing from Eurofins (I) Ltd., Bengaluru. The obtained sequences were verified for quality base call using the Chromas software. The sequence data were, thus, analyzed based on the peak base call and further checked using web-based tool BLASTn (National Center for Biotechnology Information, USA) to verify the homology with IBDV sequences. The retrieved sequence was stored as FASTA format, and the deduced amino acid was used for the further phylogenetic analysis. The deduced amino acid sequence was also analyzed for key amino acids for virulence and cell culture adaptation.

For the phylogenetic analysis, global IBDV VP_2_ nucleotide sequences were retrieved from the GenBank (National Center for Biotechnology Information, USA). The accession numbers for IBDV-VP_2_ nucleotide sequences obtained from GenBank for analysis were KT870148, KT884452, KJ547671, KJ547672, KJ547673, KJ547674, and KJ547670 (India); EF529700 and KT281984 (Pakistan); AY323952 and KC189836 (Malaysia); LM651367, LM651365, FJ695138, AF092943, and AY134874 (China); AY704912 (Iran), DQ927040 and DQ927042 (Israel); AJ31889 and NC004178 (Europe); AY918950 (Edgar-USA); JQ403646 and M97346 (USA);. D00499 (Canada); AY819701 (Classic STC, Canada); AF498631 (Bursin-Canada); and D10065 (Variant DelE, USA) and for attenuated IBDV are JX424076 (Georgia-Nigeria); EU162087 (D78-USA); AF499929 (D78-Europe); AF194428 (chicken embryo fibroblast [CEF]-Adapted-Europe); and U30818 (Serotype II).

For amino acid sequence analysis, global IBDV VP_2_ sequences were also retrieved from the GenBank. The accession numbers for IBDV-VP_2_ amino acid sequences obtained from GenBank for analysis were AMQ48647, AMQ24274, AHY99592, AHY99593, AHY99594, AHY99595, and AHY99591 (India); ABP88932 and ALD83714 (Pakistan); AAP85292 (Malaysia); CDW92045, CDW92043, AAD23373, and AAM97561 (China); AAU05319 (Iran), ABI52864 and ABI52866 (Israel); AAF16082, NP_690838, and AAF16082 (Europe); AAY16546 (Edgar-USA); AFI41891 and AAA52086 (USA); AAV68389 (STC-USA); BAA00954 (DelE-USA); BAA00391 (STC-Canada); and AAM21064 (Bursin-Canada) and for attenuated IBDV are AFU10473 (Georgia-Nigeria); ABW04864 (D78-USA); AAO15768 (D78-Europe); and avirulent AAB22968 (Serotype II).

The nucleotide and deduced amino acid sequences were analyzed using online and stand-alone nucleotide analysis software, namely Chromas, BLAST (NCBI, USA), Bioedit, and Clustal W alignment programs. Phylogenetic trees were constructed using the MEGA7 package. The distance matrices were constructed using Kimura two-parameter model and trees were constructed using the neighbor-joining algorithm. The dataset was resampled 1000 times using the bootstrap method for nucleotide sequences. For amino acid sequences, the evolutionary tree was inferred using the neighbor-joining algorithm based on the Poisson correction model and data resampling for 1000 times using bootstrap method [23-25].

## Results

**Figure-1:**
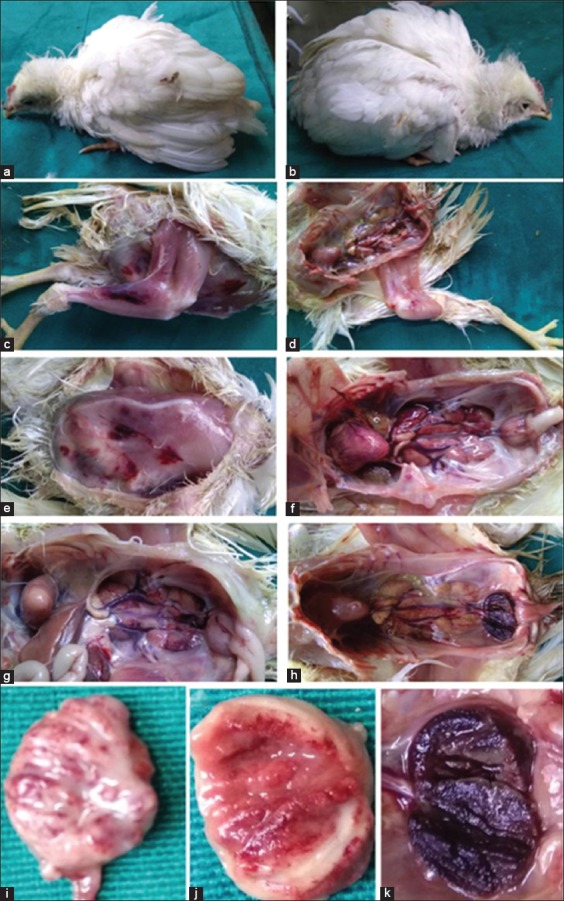
Symptoms and lesions of infectious bursal disease-suspected flocks (a and b) ruffled feathers, depression, and droopy appearance, (c-e) hemorrhages on thigh and breast muscles, (f and g) enlargement of bursa and nephritis, (h-k) bursal lesions.

The adaptation and molecular characterization of IBDV field isolate involved the investigation from postmortem lesions to its genetic and epidemiological analysis. The suspected birds showed trembling, ruffled feathers, depression, and droopy appearance. On postmortem of mortality, they showed lesions such as hemorrhages on thigh muscles, swollen kidneys, and edematous and hemorrhagic bursa (Figure-1). These symptoms and lesions were indicative of infectious bursal disease.

**Figure-2:**
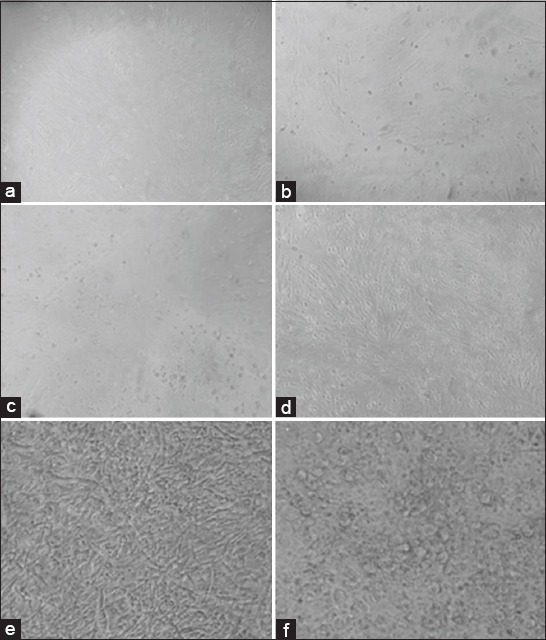
Primary chicken embryo fibroblast cells (a) uninfected, (b) 24 h post infection (PI), (c) 36 h PI, (d) 72 h PI at Passage No. 6, (e) uninfected, (f) infectious bursal disease virus-infected cells showing rounding, aggregation, vacuolation, and detachment 48 h PI at Passage No. 7.

In CEF, several CPEs, namely rounding of cells, aggregation, vacuolation, and detachment of the cells were recorded after 72 h postinoculation on sixth passage in four samples. On seventh passage, cells started showing progressive CPE at 36-48 h postinfection (Figure-2). This field isolate was designated as IBDV, PS, Nagpur, India. The chicken embryo adapted vaccine virus was kept as positive control and showed the development of CPE on third blind passage. The uninfected control CEF was maintained at every passage level and showed no CPE.

**Figure-3:**
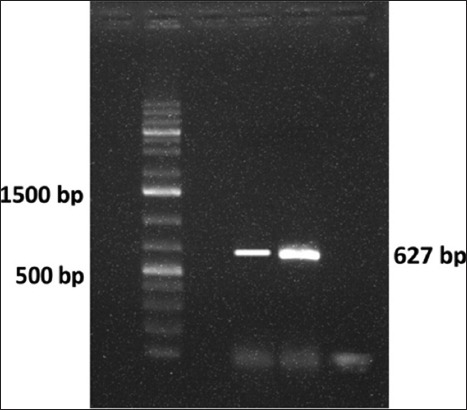
Infectious bursal disease virus (IBDV) VP_2_ gene amplicon 627 bp (Lane 2: Marker, Lane 4: VP_2_ (IBDV, PS, Nagpur, India), Lane 5: IBDV-Positive control, Lane 6: Negative control).

The monolayer showing IBDV-specific CPE was harvested with supernatant fluid and subjected for RNA extraction followed by RT-PCR using primers targeting VP_2_ structural gene hypervariable region from position 661 to 1288 on segment A of IBDV genome. The RT-PCR showed amplification of 627 bp amplicon specific to VP_2_ gene fragment (Figure-3) which confirmed successful adaptation and isolation of IBDV using CEF.

**Figure-4:**
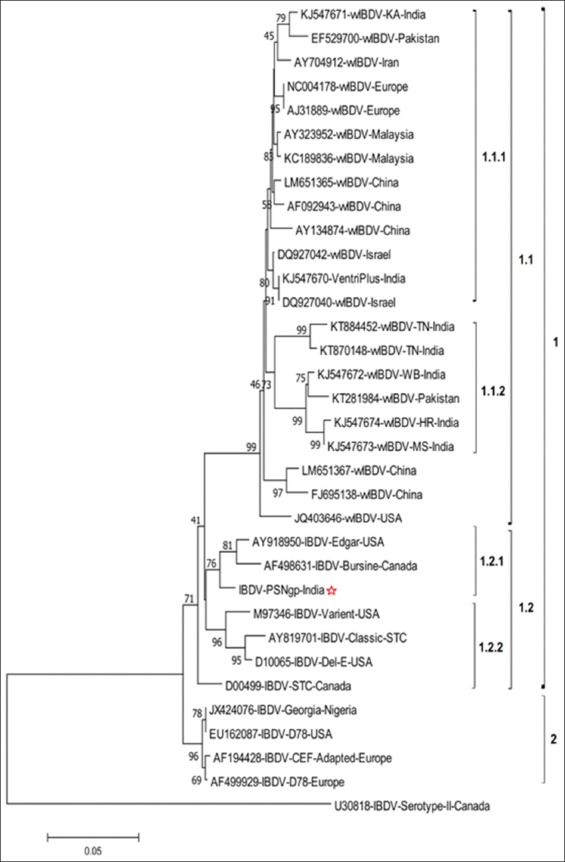
Phylogenetic tree constructed with MEGA 7.0, illustrating relationship among infectious bursal disease virus (IBDV), PS, Nagpur, India and 33 global IBDV nucleotide sequences from different geological origins. The tree was generated using neighbor-joining algorithm based on pairwise nucleotide differences in VP_2_ hypervariable region after constructing distance matrices using Kimura two-parameter model. The percentage of replicate trees in which the associated taxa clustered together in the bootstrap test (1000 replicates) is shown below the branches.

The nucleotide sequence of VP_2_ of cell culture adapted field IBDV (IBDV, PS, Nagpur, India) position from 661 to 1288 was aligned and compared with nucleotide sequences of VP_2_ hypervariable region from 33 global virulent and attenuated isolates including nine South Asian IBDVs and a serotype 2 of IBDV available on NCBI. The BLASTn results revealed that the nucleotide sequence of cell culture adapted IBDV had 98% nucleotide identity with Bursin strain (AF498631) followed by 97%, 96%, and 92-94% nucleotide identity with Edgar strain (AY918950), Ventri-Plus vaccine virus (KJ547670), and other Indian vvIBDVs, respectively (E value 0.0). The phylogenetic analysis carried out using MEGA7 package revealed serotype 2 IBDV as outliner in the phylogenetic tree (Figure-4). All the serotype 1 IBDVs were placed in a single group which was branched into two major clades. The mix population of vv, virulent, classic, and antigenic variant IBDV strains was placed in the first major clade while attenuated strains were placed in the second major clade. The IBDVs from the first major clade showed further branching into two clades. The first clade comprised all the global vvIBDV isolates. The Asian and European vvIBDVs were placed in the first subclade while South Asian vvIBDVs formed the second subclade. The second clade consisted mix population of classic, virulent, and antigenic variant IBDVs along with the IBDV field isolate (IBDV, PS, Nagpur, India) under investigation. It was placed with Bursin (AF498631) and Edgar (AY918950) strains in a distinct subclade.

**Figure-5:**
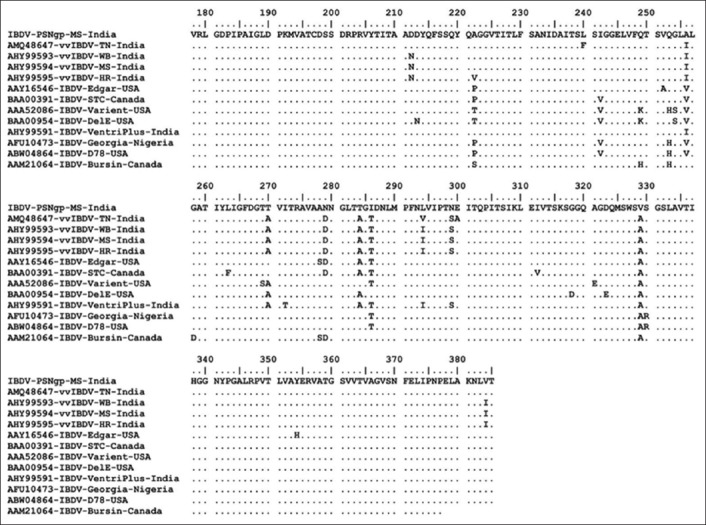
Alignment of deduced amino acid sequences for the hypervariable region of VP2 gene of Indian very velogenic, classical, variant, and attenuated strains of infectious bursal disease virus (IBDVs) with IBDV, PS, Nagpur, India. Identity of the aligned amino acids is shown by dots and differences by single letter.

**Figure-6:**
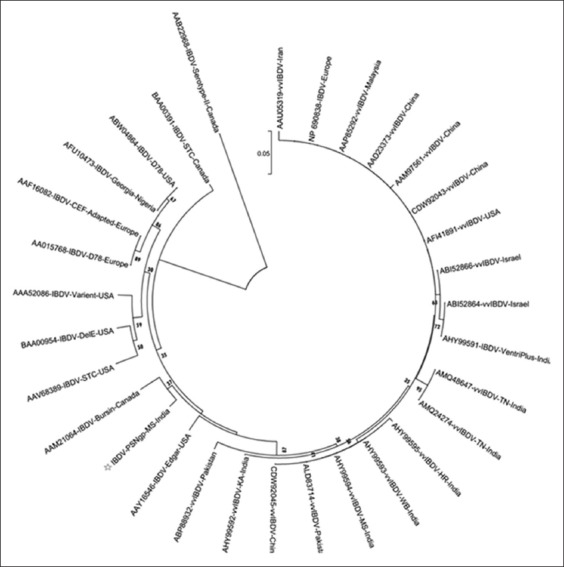
Phylogenetic tree constructed with MEGA7, illustrating relationship among infectious bursal disease virus (IBDV), PS, Nagpur, India and 30 global IBDVs based on deduced amino acid sequences from different geological origins. The tree was generated using neighbor-joining algorithm and evolutionary distances were computed using the Poisson correction method. The percentage of replicate trees in which the associated taxa clustered together in the bootstrap test (1000 replicates) is shown below the branches.

The deduced amino acid sequence of hypervariable VP_2_ fragment of cell culture adapted field IBDV (IBDV, PS, Nagpur, India) position from 178 to 385 was considered for analysis. The amino acid sequence analysis revealed the major amino acid changes at different locations as depicted in Figure-5. The isolate showed A222, I242, Q249, Q253, A256, T270, N279, T284, I286, L294, N299, and V329. The phylogenetic analysis of deduced amino acid sequence of VP_2_ is shown in Figure-6. The phylogenetic tree indicated clustering of all the vvIBDVs in clade 1 and mix population of classical, virulent, antigenic variant, and attenuated IBDVs in clade 2. The IBDV, PS, Nagpur, India, was placed with Bursin and Edgar strains. The serotype 2 IBDV was placed as outliner in the phylogenetic tree.

## Discussion

Isolation of pathogen is considered as gold standard for diagnosis. The cell culture adaptation was aimed to confirm the presence of replicating IBDV in bursal samples and to study its multiplication cycle in CEF. The present investigation states about the successful adaptation of field IBDV (IBDV, PS, Nagpur, India) on primary CEF cells. The adaptation of IBDV is difficult in cell culture system. It took several blind passages for adaptation on CEF as earlier reported by McFerran *et al*. [26]. The vaccine virus showed CEF adaptation at third passage level while the IBDV, PS, Nagpur, India could be successfully adapted at seventh serial passage level. The CPEs were similar in vaccine virus, and field isolate such as rounding, detachment, and vacuolation of the cells from CEF monolayer. The similar CPEs were reported earlier [27]. The present investigation also reports multiplication cycle of 36–42 h in CEF which is longer (10–36 h) than in chicken embryo kidney cells and shorter (48 h) than in Vero cells [28,29] which could be due to the adaptation of the field virus on the cells.

Further confirmation by VP_2_ gene-based RT-PCR indicated the specific amplicon of 627 bps which is in accordance with a previous report [22]. The VP_2_ gene is very specific to IBDV, and several researchers used it for the detection of IBDV from field samples by RT-PCR [30,31].

The molecular epidemiology of IBDV is based on VP_2_ nucleotide sequences from different countries and geographical locations. The hypervariable region of VP_2_ gene of IBDV is commonly utilized to differentiate and classify IBDV isolates [30]. The 43 kDa VP_2_ is the major structural protein of IBDV and possesses the antigenic region for the induction of neutralizing antibodies [32]. The present investigation revealed that the CEF adapted field isolate shared 98% identity with Bursin (AF498631) isolate reported from Canada and 97% with Edgar challenge strain (AY918950), whereas only 96% nucleotide identity was recorded with Ventri-Plus vaccine strain (KJ547670) and 92%-94% identity with other Indian isolates.

The hypervariable VP_2_ amino acid sequence of the IBDV, PS, Nagpur, India, isolate shared conserved amino acids reported in vvIBDV isolates at positions A222, I242, and Q253 and typically reported in classic, antigenic variant, and attenuated strains at amino acids positions T270, N279, T284, L294, and N299. It showed Q249, T284, and I286 as reported in variant and classic IBDVs [33] except amino acid at G254. The IBDV, PS, Nagpur, India, also showed unique amino acid substitution at position A329V which was not observed in any other IBDVs. The amino acid substitutions were recorded at several other positions in hypervariable region of VP_2_ [34]. The analysis also revealed that the isolate possesses key amino acids required for cell culture replication at N279 and T284 but not at Q253. It has been reported that the amino acids at positions H253, N279, and T284 in the VP_2_ protein are involved in cell tropism [33,34], and amino acid at position 253 is critical for virulence [35]. It is suggested that the amino acid Q253 may be involved for *in vivo* infection, whereas H253 for cell-culture adaptation [36]. The residues at positions N279 and T284 have been suggested for cell culture replication, whereas D279 and A284 for virulence [33,34,37]. The putative amino acids at position A222, I242, Q253, I256, D279, A284, I294, and S299 are suggested as key virulence markers in VP_2_ and are conserved in vvIBDV [33,38-40]. Out of these, only three amino acids (A222, I242, and Q253) were reported in IBDV, PS, Nagpur, India, isolate. The amino acids at positions Q253 and T284 are similar to the IBDV variant pathogens [41]. This was further signified with the finding where the researchers have claimed that the glutamine at positions 249 and 253 might have been involved in increased virulence of the present isolate [42], indicating that the isolate in the present study might be of virulent nature and on adaptation showing attenuation with critical amino acid changes.

The phylogenetic investigation based on neighbor-joining algorithm using Kimura two-parameter and 1000 bootstrap test of nucleotide sequences indicates clustering of IBDV based on virulence and geographical locations. The classical, virulent, antigenic variant, and attenuated IBDV formed a separate clade from vvIBDVs. The virulent IBDVs from different geographical locations showed divergence in the same major clade [43]. The vaccine virus (AHY99591) being used in the region was closely placed with vvIBDV Israeli isolates (DQ927040 and DQ927042) as a separate subcluster in virulent IBDV cluster. The IBDV field isolate (IBDV, PS, Nagpur, India) was placed in a major cluster along with AF498631 (Bursin-Canada) and AY918950 (Edgar strain) which was hot and challenge strain used in IBDV research [44]. The indication of placing of IBDV, PS, Nagpur, India, field isolate with Edgar strain suggested that this isolate has potential to cause morbidity and mortality and is one of the divergences of such hot strain circulating in the Nagpur region. This CEF adapted field isolate may have been originated from classical IBDV circulating in the field. The results are also indicative of the existence of IBDV strains with varying virulence in the country. We speculate that the use of low pathogenic, mild, and hot strains as live vaccines might result into emergence of such IBDV strain in this region of Maharashtra state. The similar observations have been documented showing evolution of the new IBDV variants with low, mild, and vvIBDVs in the field due to the coexistence of field strains and live attenuated vaccine strains being used for boosting protective immune response [45,46].

Further investigation at full genomic level will be helpful to reveal amino acid changes which might be indicative of new strain. Based on the findings, it is observed that the cell culture adapted field IBDV isolate investigated in the present study does not reveal the full nucleotide signature of vvIBDV as well as vaccine strains. Hence, we can conclude that it might not belong to vvIBDVs of Indian origin and the vaccine strain used in the region, suggestive of possibility of virus evolution due to the coexistence of circulating field strains and live attenuated hot strains, resulting into morbidity and mortality, warranting need for safer protective vaccines, and implementation of stringent bio-security measures to minimize loss to poor and marginal poultry farmers in developing countries like India where poultry husbandry is being considered as one of the alternatives to support farmer’s livelihood.

## Conclusion

The IBDV field isolate does not reveal the full nucleotide sequence signature of vvIBDV as well as vaccine strains. Hence, we can conclude that it might not belong to vvIBDVs of Indian origin and the vaccine strain used in the region. This may be suggestive of the evolution of the IBDV in the field due to the coexistence of circulating field strains and live attenuated hot strains, resulting into morbidity and mortality, warranting the need for safer protective vaccines, and implementation of stringent bio-security measures to minimize loss to farmers.

## Authors’ Contributions

SPA carried out experimental work, analyzed the data, and wrote the manuscript. PAT analyzed the data and supervised the experiment. NVK, SPC, SWB, and VCI supervised the experiment. JAK participated in the organization of laboratory work. All authors have read and approved the manuscript.
